# Would early removal of indwelling catheter effectively prevent urinary retention after hip fracture surgery in elderly patients?

**DOI:** 10.1186/s13018-019-1360-1

**Published:** 2019-09-18

**Authors:** Dae-Kyung Kwak, Chul-Young Oh, Jeong-Seop Lim, Hyung-Min Lee, Je-Hyun Yoo

**Affiliations:** 1 Department of Orthopaedic Surgery, Hallym University Sacred Heart Hospital, Hallym University School of Medicine, 896 Pyeongchon-Dong, Dongan-gu, Anyang, 431-070 South Korea; 2 Department of Urology, Hallym University Sacred Heart Hospital, Hallym University School of Medicine, Anyang, South Korea

**Keywords:** Elderly patients, Hip fracture surgery, Postoperative urinary retention, Indwelling catheterization

## Abstract

**Background:**

This study aimed to investigate the incidence and risk factors of postoperative urinary retention (POUR) among elderly patients who underwent hip fracture surgery and to evaluate the effect of indwelling catheterization on the occurrence of POUR.

**Materials and methods:**

From January 2012 to January 2015, consecutive patients aged over 70 years who underwent hip fracture surgery were enrolled in this study. All patients underwent indwelling catheterization due to voiding difficulty upon admission. Demographic data, perioperative variables, and postoperative duration of patient-controlled analgesia and indwelling catheterization, postoperative complications, and mortality were collected. The incidence of POUR was investigated, and the risk factors related to POUR were analyzed using a logistic regression analysis. The cutoff value for the timing of catheter removal was determined using receiver operating characteristic (ROC) curve analysis.

**Results:**

POUR developed in 68 patients (31.8%) of the 214 patients. Of these, 24 (35.3%) were male. The indwelling catheter was left in place for an average of 3.4 days (range, 0–7 days) postoperatively. A significant difference was noted in gender and duration of indwelling catheterization between patients with POUR and without. The cutoff value for the timing of catheter removal as determined by ROC curve analysis was 3.5 postoperative day with 51.4% sensitivity and 71.5% specificity. Multiple logistic regression revealed that the duration of the indwelling catheter [odds ratios (OR), 0.31; *p* = 0.016)] and male gender (OR, 2.22; *p* = 0.014) were independent risk factors related to the occurrence of POUR.

**Conclusions:**

The significant risk factors of POUR among elderly patients undergoing hip fracture surgery were early indwelling catheter removal and male gender. Therefore, early removal of indwelling catheter in elderly patients following hip fracture surgery may increase the risk of POUR, especially in male patients.

## Introduction

Postoperative urinary retention (POUR) is a well-known complication of various surgical procedures, especially in elderly patients, with a reported prevalence of 5–70% [1, 2]. Urinary retention is a relatively common complication among patients with hip fracture, and its incidence may reach to 82% preoperatively and 56% postoperatively, with much variation in previous literature [3]. The consequences of POUR can be more complicated with potential local and distant sequelae. Apart from causing unnecessary pain and discomfort to patients, bladder dysfunction can lead to urinary tract infection (UTI) or bacteremia, thereby acting as a source of infection for any implants and sepsis. POUR has been associated with the risk of bladder overdistension, which may produce permanent detrusor dysfunction and increase the post-void residual volume (PVR). Repeated attempts to catheterize the bladder can predispose patients to UTI. Nevertheless, less attention is paid to POUR compared with postoperative wound infections, bleeding, ileus, pneumonia, and delirium [4].

POUR is managed by intermittent or indwelling catheterization [5]. However, Scholten et al. [6] reported that most POUR patients were discharged with intermittent or indwelling catheter because the condition was not resolved at an early stage. These problems may lead to passivity, resignation, and a bedridden state [7]. One study reported that 32% of over 110,000 patients with hip fracture discharged to “skilled nursing facilities” had a urinary catheter in place, and this increased the 30-day risk for re-hospitalization for UTI and death [8]. Therefore, prevention of POUR is of paramount importance in elderly patients.

Pain is a typical stimulus to activate the sympathetic nervous system [9, 10]. Activation of the sympathetic nervous system causes the bladder to increase its capacity and stimulates the internal urinary sphincter to remain tightly closed [11]. Likewise, most elderly patients with hip fracture have experienced voiding difficulty caused by immobility, severe pain related to fracture, and subsequent contraction of the internal sphincter of the bladder [9, 12]. These problems may lead to perioperative delirium and POUR, which have a negative effect on the surgical outcomes [12]. Therefore, indwelling catheterization has been performed during admission for elderly patients with hip fracture in our institute to prevent POUR due to pain and immobility perioperatively.

However, there have been few studies investigating the association of the time of urinary catheter removal and POUR among elderly patients who underwent hip fracture surgery. The aim of the current study was to investigate the risk factors including the timing of indwelling catheter removal and incidence of POUR following hip fracture surgery in elderly patients.

## Materials and methods

This study was conducted after obtaining ethical approval from our institutional review board (2015-I032). All available electronic medical records were retrospectively reviewed to identify patients aged ≥ 70 years who underwent hip fracture surgery between January 2012 and January 2015. Intramedullary nailing was performed for intertrochanteric fracture, and hip arthroplasty was performed for femoral neck fracture. Patients with a history of urinary retention or urinary catheterization before admission and those who were diagnosed with benign prostatic hyperplasia (BPH), operated for that, or unable to ambulate postoperatively, were excluded in this study.

Indwelling catheterization was performed routinely for all patients just after admission. The catheter was removed considering the possibility of self-voiding when patients were able to stand and ambulate with a walker postoperatively. Patients who were unable to void 4 h after urinary catheter removal were identified, and the PVR urine volume was measured using an ultrasound bladder scan or urethral catheterization to detect POUR. POUR was diagnosed if the PVR volume was > 400 mL. Indwelling re-catheterization was performed for patients confirmed with POUR by a urologist. Those with unresolved POUR during their hospital stay were eventually discharged with an indwelling catheter. Routine follow-up in the urology outpatient clinic was planned 2 weeks after discharge, and oral medications were prescribed if indicated.

According to our institutional protocol, the patients were instructed to walk under tolerable weight-bearing conditions with an assistive device (walker or cane) from the second postoperative day to 4–6 weeks postoperatively. All patients received the same analgesics according to our institutional protocol for postoperative pain control: Celebrex (celecoxib; 200 mg every 12 h), Ultracet (acetaminophen; 325 mg/tramadol HCl; 37.5 mg every 6 h), and patient-controlled analgesia (PCA). For breakthrough pain, all patients received intravenous ketorolac (30 mg every 6 h as needed) in the postoperative period.

Demographic data on the age, gender, body mass index (BMI), bone mineral density (BMD), American Society of Anesthesiologists (ASA) score, and underlying diseases were obtained. As perioperative variables, the anesthesia type, operation time, amount of intraoperative infused fluids and urine output, estimated blood loss (EBL), time to rehabilitation after surgery, and type of PCA infusion were collected. In addition, the length of hospital stay, duration of PCA infusion and of indwelling catheter after surgery, and the occurrence of UTI were investigated.

The EBL was calculated according to the Gross formula as shown below.

Prediction of blood volume [13]:

$$ {\displaystyle \begin{array}{c}\mathrm{Male}:604+0.0003668\times {\left[\mathrm{Height}\ \left(\mathrm{cm}\right)\right]}^3+32.2\times \mathrm{weight}\ \left(\mathrm{kg}\right)\\ {}\mathrm{Female}:183+0.000356\times {\left[\mathrm{Height}\ \left(\mathrm{cm}\right)\right]}^3+33\times \mathrm{weight}\ \left(\mathrm{kg}\right)\end{array}} $$

EBL calculation method [14]:

$$ \mathrm{EBL}=\mathrm{blood}\ \mathrm{volume}\times \left({\mathrm{Hematocrit}}_{\mathrm{preoperative}}-{\mathrm{Hematocrit}}_{\mathrm{day}\ 5\ \mathrm{postoperative}}\right)+\mathrm{mL}\ \mathrm{of}\ \mathrm{transfused}\ \mathrm{red}\ \mathrm{blood}\ \mathrm{cell} $$

### Statistical analysis

Basic descriptive statistical analyses were used to describe the study population. Values were subjected to averaging or percentages with the use of SPSS version 17.0 (SPSS Inc., Chicago, IL, USA). Student’s *t* test was used for continuous variables and chi-square test for categorical variables between the two groups with POUR and without. A *p* value < 0.05 was considered as statistically significant. Variables with *p* value < 0.05 were incorporated into a forward, stepwise multivariate regression analysis to determine the risk factors for POUR. Odds ratios (OR) were obtained with 95% confidence intervals (CI) for independent risk factors. The receiver operating characteristic (ROC) curves were utilized to determine the area under the ROC curve (AUC) and cutoff value for the timing of catheter removal.

## Results

**Fig. 1:**
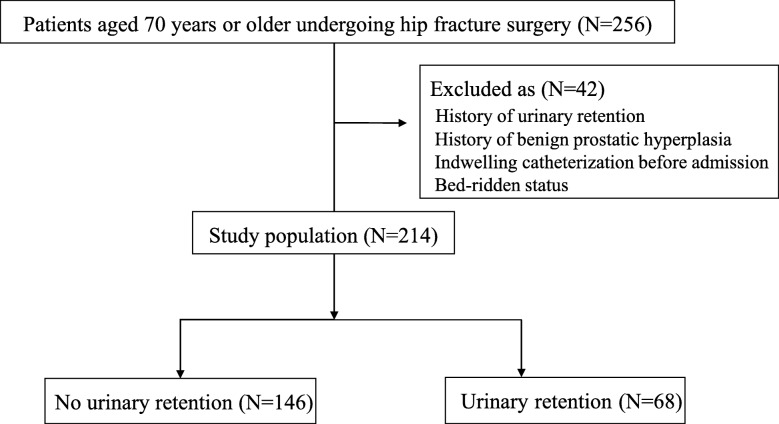
Flowchart demonstrating patient selection

During the study period, a total of 256 patients were identified. Of those, 214 patients with the mean age of 78.7 years (range, 70–99 years) were enrolled in the final cohort (Fig. 1). Of 214 patients, 162 (74.3%) were female. Seventy-seven patients (36.0%) had underlying diseases affecting POUR: diabetes mellitus, brain and spinal disease, or neurogenic bladder. General anesthesia was performed in 104 patients (48.6%) and spinal anesthesia in 110 patients (51.4%). The mean operation time was 85.0 min (range, 35–170 min). The mean amount of intraoperative infused fluid was 1181.8 mL (range, 130–2450 mL) while the mean EBL volume was 522.9 mL (range, 50–1200 mL). The mean duration of PCA was 2.2 days (range, 1–5 days), and the indwelling catheter was left in place for an average of 3.4 days (range, 0–7 days). The mean length of hospital stay was 24.1 days (Table 1).

**Table 1:** Data of 214 patients over 70 years old with hip fractures

Variables	Mean (range)
Age (years)	78.7 (70–99)
Gender (men : women)	52:162
Body mass index (kg/m^2^)	22.3 (13–33)
Bone mineral density (*T*-score)	− 2.6 (− 5.3 to − 0.3)
ASA score	2.9 (2–4)
II	37
III	155
IV	22
Number of underlying disease*	0.7 (0–2)
Anesthesia (general : spinal)	104:110
Operation time (minutes)	85.0 (35–170)
Amount of intraoperative fluid infused (mL)	1181.8 (130–2450)
Amount of estimated blood loss (mL)	522.9 (50–1200)
Time to rehabilitation after surgery	
POD 2	157
POD 3	48
POD 4	6
≥ POD 5	3
PCA (intravenous : epidural)	198:16
Duration of PCA infused (days)	2.2 (1–5)
Duration of Foley catheter indwelling (days)	3.4 (0–7)
Duration of hospitalization (days)	24.1 (9–105)

*ASA* American Society of Anesthesiologists, *POD* postoperative day, *PCA* patient-controlled analgesia

*Underlying disease includes diabetes mellitus, brain disease, spinal disease, or neurogenic bladder

Among 214 patients, POUR was observed in 68 patients (31.8%), which is composed of 44 females (64.7%) and 24 males. A significant difference was noted in the gender and postoperative duration of indwelling catheterization (*p* = 0.016 and *p* = 0.004, respectively) between the two groups with POUR and without. More male patients developed POUR, and postoperative duration of the indwelling catheter was shorter in patients with POUR than in those without POUR (Table 2). No significant differences were observed in the age, BMI, ASA score, BMD, fracture site, underlying diseases, anesthesia type, operation time, volume of intraoperative urine output and EBL, transfusion amount, time to rehabilitation after surgery, type and duration of PCA, and duration of hospitalization between both groups.

**Table 2:** Comparison data between the two groups

	POUR (−) (*n* = 146)	POUR (+) (*n* = 68)	*p* value
Age (years)	78.6 *±* 6.6	78.7 *±* 6.7	0.898
Gender (male : female)	28:118	24:44	0.016
Body mass index (kg/m^2^)	22.2 *±* 3.0	22.3 *±* 3.6	0.470
ASA score			0.117
II	26	11	
III	105	50	
IV	15	7	
Bone mineral density (*T*-score)	− 2.7 ± 1.0	− 2.6 ± 0.8	0.563
Fracture site (neck : trochanter)	54:92	25:43	1.000
Patients with underlying diseases* (%)	50 (34.2)	27 (39.7)	0.072
Anesthesia (general : spinal)	70:76	34:34	0.883
Operation time (minutes)	84.9 *±* 27.0	85.0 *±* 32.0	0.973
Amount of intraoperative urine output (cc)	201.0 ± 174.8	202.3 ± 170.7	0.959
Amount of estimated blood loss (mL)	504.8 *±* 199.6	561.8 *±* 262.5	0.115
Amount of transfusion (units)	2.8 ± 1.9	3.2 ± 2.0	0.250
Time to rehabilitation after surgery			0.854
POD 2	107	50	
POD 3	33	15	
POD 4	4	2	
≥ POD 5	2	1	
PCA (intravenous : epidural)	137:9	61:7	0.402
Duration of PCA infused (days)	2.2 *±* 0.6	2.2 ± 0.7	0.400
Duration of Foley catheter indwelling after surgery (days)	3.7 *±* 3.3	2.8 *±* 1.2	0.004
Duration of hospitalization (days)	13.8 *± 5.7*	14.7 *±* 5.5	0.657
Urinary tract infection (%)	5 (3.4%)	6 (8.8%)	0.107

Values are presented as mean (*±* standard deviation)

*ASA* American Society of Anesthesiologists, *POD* postoperative day, *PCA* patient-controlled analgesia

*Underlying disease include diabetes mellitus, brain disease, spinal disease, and neurogenic bladder

Among 214 patients, only 11 patients (5.1%) developed UTI, which consisted of 6 patients with POUR and 5 patients without POUR. The incidence of UTI was higher in patients with POUR (6/68, 8.8%) than in those without POUR (5/146, 3.4%). However, there was no significant difference between both groups.

**Fig. 2:**
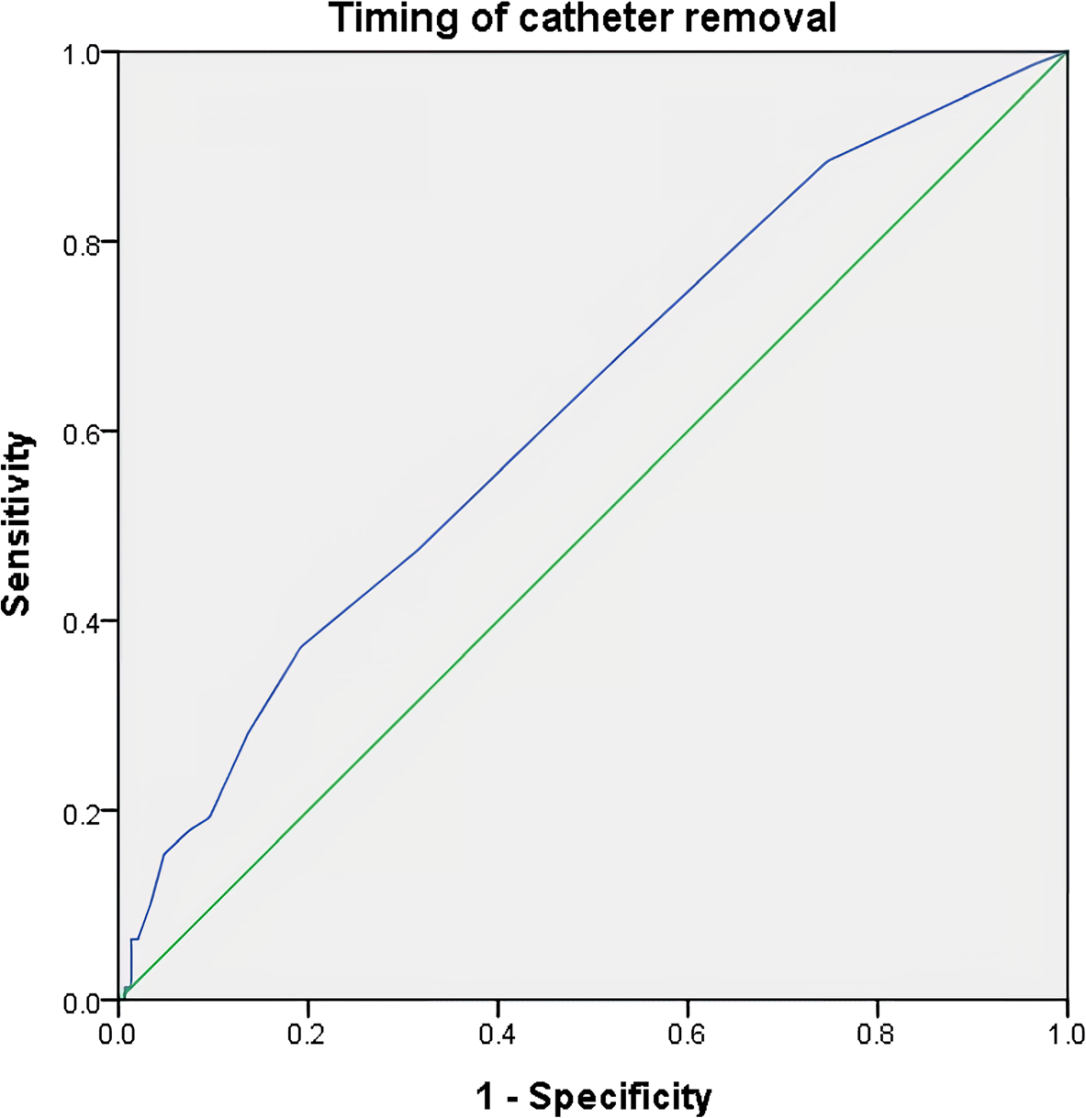
Receiver operating characteristic curves for the timing of catheter removal

Multiple logistic regression analysis revealed that the indwelling period of urinary catheter (per 1-day increase, OR = 0.31, 95% CI 0.14–0.78; *p* = 0.016) and male gender (OR = 2.22, 95% CI 1.17–4.22; *p* = 0.014) were risk factors of POUR. ROC analysis was used to define the cutoff value for the appropriate timing of indwelling catheter removal. The AUC for the timing of catheter removal was 0.63 (95% CI 0.56–0.70), and the cutoff value was 3.5 days after surgery (sensitivity, 51.4%; specificity, 71.5%) (Fig. 2).

## Discussion

Several reports revealed that POUR is highly prevalent especially in elderly patients with hip fracture [15–17]. Such condition is likely to lead to an increase in the length of hospital stay and morbidity rate. Recently, Golubovsky et al. reported that POUR was significantly associated with an increased risk of UTI and sepsis, increased length of hospital stay, and discharge to a skilled nursing facility [18]. It is of paramount importance to prevent POUR in these patients. Most elderly patients with hip fracture have experienced voiding difficulty perioperatively caused by immobility, severe pain related to fracture and surgery, and subsequent contraction of the internal sphincter of the bladder [9, 12]. Therefore, perioperative indwelling catheterization may be an effective alternative to reduce bladder dysfunction and subsequent POUR in elderly patients with hip fracture [16, 19]. Therefore, we have performed indwelling catheterization upon admission in all elderly patients undergoing hip fracture surgery. Previous studies reported that the frequency of UTI among elderly patients with hip fracture was between 12 and 52% [20–22]. Early urinary catheterization may be a risk factor of UTI. However, in this study, only 11 (5.1%) of elderly patients with perioperative indwelling catheterization developed UTI and the incidence was lower than other studies not performing routine indwelling catheterization at admission in elderly patients with hip fracture. We believe that routine indwelling catheterization in elderly patients with hip fracture is safe without increased risk of UTI and subsequent complications.

Previous studies have shown a broad spectrum of the incidence of POUR (7–84%) because of variable diagnostic criteria and risk factors [1, 23]. In the current study, the incidence of POUR among elderly patients undergoing hip fracture surgery was 31.8%. The known risk factors of POUR are age > 60 years, spinal anesthesia, duration of surgery > 120 min, diabetes, UTI, delirium, anticholinergic medication, analgesics, and constipation [15, 21, 24]. A number of studies have demonstrated that older patients have increased risk of POUR compared to younger patients [18, 25, 26]. However, this study included only elderly patients ≥ 70 years undergoing hip fracture surgery. As a result, the age had no significant impact on the occurrence of POUR among elderly patients ≥ 70 years. Several studies reported that spinal anesthesia is correlated with a high incidence of POUR [6, 27, 28]. However, spinal anesthesia had no significant impact on the occurrence of POUR in the present study because no significant difference was noted in the anesthesia type between both groups. Systemic opioid can affect the occurrence of POUR by inhibiting the release of acetylcholine from the parasympathetic sacral neurons that control detrusor contractility [17]. In our study, there was no significant difference between the two groups in terms of the type and duration of PCA infused. Therefore, opioid administration was not also associated with the occurrence of POUR. However, the overall incidence of POUR in this study might be higher than that of other studies because PCA was performed in all patients. Sex-specific diseases such as BPH may be a possible factor with a significant difference in the gender [29]. Tammela et al. [30] reported that the incidence of POUR was higher in males (4.7%) than in females (2.9%) out of 5220 surgical patients. A prospective cohort study that included males and females undergoing lower limb arthroplasty under spinal anesthesia reported that male sex and old age were significant risk factors of POUR [29]. In the study by Griesdale et al. [31], male gender was also identified as a significant risk factor of POUR following total joint arthroplasty. The present study was similar to the previous studies that identified male gender as a significant risk factor. The incidence of POUR in the current study was higher in male patients (46.2%) than female patients (27.2%). We excluded male patients who were diagnosed with or operated for BPH prior to the injury to rule out this risk factor. However, some patients with undiagnosed BPH might be included.

Our study showed that the indwelling period of postoperative catheterization affected the occurrence of POUR. Early removal of indwelling catheter is generally recommended to prevent catheter-related UTI and other complications [32]. However, early removal of indwelling catheter increased the risk of POUR following hip fracture surgery in elderly patients as shown in this study. The incidence of UTI in our study performing indwelling catheterization at admission in all patients was lower than other studies. Knight et al. [33] reported that catheterization performed 24–48 h following lower limb arthroplasty reduces POUR without increasing the risk of UTI. Meanwhile, most recent studies suggest that routine preoperative indwelling catheterization is not warranted, and intermittent catheterization is recommended to treat POUR in patients undergoing total joint arthroplasty [34, 35]. Nonetheless, these studies are limited to arthroplasty surgery while this study aimed at only elderly patients ≥ 70 years undergoing hip fracture surgery. Patients undergoing total joint arthroplasty are relatively young compared to hip fracture patients, and postoperative ambulation and rehabilitation in arthroplasty patients can be performed earlier and more rapidly because they do not have severe pain like elderly patients those with hip fracture. Therefore, we believe that the approach to prevent and treat POUR in elderly patients with hip fracture should be different from that in arthroplasty patients, especially in terms of use of indwelling catheter and the time of catheter removal.

Our findings show that the duration of the indwelling catheter was shorter in patients with POUR than in patients without POUR. Although unnecessary indwelling catheterization should be avoided, removal of indwelling catheter should be performed considering the patient’s general condition and ambulation status. Early routine removal of indwelling catheter without considering the patient’s condition will more likely cause POUR and eventually increase the duration of indwelling catheterization, leading to subsequent complications and poor functional outcomes in fragile elderly patients. In most elderly patients with hip fracture, several days are needed for self-voiding after starting postoperative rehabilitation such as tilt table exercise and parallel bar walking. Postoperative rehabilitation and self-voiding may need more time after hip fracture surgery in most elderly patients with comorbidities. Riehmann et al. reported that the mean peak flow rates of voiding were lower and the PVR volume was larger in the recumbent position than the standing in elderly patients with hip fracture [36]. Therefore, early ambulation and rehabilitation were crucial to obtain satisfactory functional outcome and prevent POUR. It would be also of paramount significance that the operating surgeon identify elderly patients with increased risk of POUR to do so.

This study has several limitations. First, this was a retrospective study design. Second, there was no comparison with a control group without indwelling catheterization. Third, although the cutoff value for the timing of indwelling catheter removal was evaluated in this study, the sensitivity and specificity were relatively low.

However, this is the first study to investigate POUR in fragile elderly patients ≥ 70 years with hip fracture. Second, we could exclude other known risk factors affecting the occurrence of POUR between the two groups. Third, we compared underlying diseases such as diabetes mellitus, brain disease, spinal disease, or neurogenic bladder that could affect the occurrence of POUR between both groups. Finally, this study provided the cutoff value for the timing of indwelling catheter removal to prevent POUR in elderly patients with hip fracture. However, prospective studies with larger cohort are needed to substantiate our results in the future.

## Conclusion

The incidence of POUR in elderly patients ≥ 70 years undergoing hip fracture surgery was 31.8%. The significant risk factors were male gender and early removal of indwelling catheter after surgery. Therefore, early removal of indwelling catheter within 3 days following hip fracture surgery in elderly patients may increase the risk of POUR, especially in male patients.

## Data Availability

The datasets used and/or analyzed during the current study are available from the corresponding author on reasonable request.

## Abbreviations

ASA: American Society of Anesthesiologists
AUC: Area under the ROC curve
BMD: Bone mineral density
BMI: Body mass index
BPH: Benign prostatic hyperplasia
CI: Confidence intervals
EBL: Estimated blood loss
OR: Odds ratios
PCA: Patient-controlled analgesia
POUR: Postoperative urinary retention
PVR: Post-void residual volume
ROC: Receiver operating characteristic
UTI: Urinary tract infection
